# Luminescence Properties and Energy Transfer in SrLa2Sc2O7 Co-Doped with Bi^3+^/M (M = Eu^3+^, Mn^4+^, or Yb^3+^)

**DOI:** 10.3390/ma15228040

**Published:** 2022-11-14

**Authors:** Tao Wang, Huifang Yang, Zhijun Wang, Panlai Li

**Affiliations:** 1 College of Science, China University of Petroleum (East China), Qingdao 266580, China; 2 Hebei Key Laboratory of Optic-Electronic Information and Materials, College of Physics Science & Technology, Hebei University, Baoding 071002, China

**Keywords:** luminescence, phosphor, energy transfer

## Abstract

Series of Eu^3+^/Mn^4+^/Yb^3+^-doped SrLa_2_Sc_2_O_7_:Bi^3+^ (SLSO: Bi^3+^) were synthesized by a high-temperature solid-state method, and the energy transfer of Bi^3+^→Eu^3+^/Mn^4+^/Yb^3+^ was observed. Under ultraviolet radiation, a 550 nm emission peak was observed, which is attributed to Bi^3+^ occupying the Sr^2+^/La^3+^ sites. Additionally, the other peaks were found to be 615, 707, and 980 nm, which are assigned to the Re^3+^ (Eu^3+^ and Yb^3+^) and Mn^4+^ occupying two different cationic sites. An obvious energy transfer (ET) from Bi^3+^ to Eu^3+^/Mn^4+^/Yb^3+^ was observed, and the tunable color, emitting from yellow to red, was obtained; the ET efficiency was about 86.2%, 78.6%, and 27.5% in SLSO, respectively. We found that the large overlap area between the emission spectrum of the sensitizer and the excitation spectrum of the activator could produce efficient energy transfer, which provided the idea for designing experiments in the future for some highly efficient energy transfer processes.

## 1. Introduction

Recently, luminescent materials have been widely used in light-emitting diodes (LEDs), medical devices, 3D displays, temperature sensing, and other applications [1,2]. Being an essential component of LED devices, the different color emitting phosphors have been investigated and discussed; they are considered one of the biggest challenges in the field of lighting and backlighting display [3,4]. Generally, the different emitting phosphors can be achieved by energy transfer (ET) [5,6,7,8,9,10,11,12]. As is well known, Eu^2+^, Ce^3+^, and Bi^3+^ are effective sensitizers because they can emit broad bands in various hosts, depending on their 4f^6^5d^l^-4f^7^, 5d^1^-4f^l^, and ^3^P_1_-^1^S_0_ allowed transitions [13,14,15], respectively, and can transfer the energy to activators, such as rare earth ions (Re^3+^ = Eu^3+^, Sm^3+^, Tb^3+^, Yb^3+^, Nd^3+^, etc.), and achieve the different color emitting phosphors [16,17,18]. The 6s^2^ configuration of the Bi^3+^ ion is sensitive to its surroundings of the local crystal field but is more stable than other valence states such as Bi^0^, Bi^2+^, and Bi^5+^; hence, Bi^3+^ ion exhibits a variety of emissions involving ultraviolet, blue, green, yellow, and red [19,20,21]. In contrast, unique spectral features such as narrow emission bands, long luminescence lifetimes, and diverse emission states are presented by Re^3+^ ions with a 4f electronic configuration [22]. Therefore, both are considered candidates for the potential of multiple luminescent color materials. In this work, in order to improve the luminescent properties of activator Eu^3+^/Mn^4+^/Yb^3+^, we select SrLa_2_Sc_2_O_7_ as the host and Bi^3+^ as the sensitizer for multi-colored phosphors. In scandium salts, the Sc^3+^ ion does not follow the contraction law of the lanthanides and possesses a smaller ionic radius. Due to the lack of 4f electrons, it has a different electron configuration than other rare earth elements, giving it special physicochemical properties that allow Sc-based matrices to exhibit excellent photoluminescence. We, therefore, chose SrLa_2_Sc_2_O_7_ matrix crystals. In this paper, we choose the co-doping of Bi^3+^→Eu^3+^; Bi^3+^→Mn^4+^; Bi^3+^→Yb^3+^ to achieve visible to near-infrared light emission in the SrLa_2_Sc_2_O_7_ matrix, with Bi^3+^ as the sensitizer and Eu^3+^/Mn^4+^/Yb^3+^ as the activator. It is noted that SrLa_2_Sc_2_O_7_ had three cationic environments: SrO_12_, Sr/LaO_9_, and ScO_6_ polyhedrals, respectively. The emission color can turn from yellow to red and near-infrared light by the Eu^3+^, Yb^3+^, and Bi^3+^ entering into the Sr/LaO_9_ polyhedral in terms of ion valence and radius and because the Mn^4+^ ion has a small radius, which can occupy the ScO_6_ site to produce the yellow to deep red light. Moreover, the luminescence properties and the energy transfer progress of Eu^3+^/Mn^4+^/Yb^3+^/Bi^3+^ in SrLa_2_Sc_2_O_7_ are also discussed. We also explore the interaction between the sensitizer and the activator and find that the large overlap area between the emission spectrum of the sensitizer and the excitation spectrum of the activator produces efficient energy transfer, which provides the idea for designing experiments in the future for some highly efficient energy transfer processes.

## 2. Materials and Methods

A series of SrLa_2_Sc_2_O_7_:0.06Bi^3+^, Re^3+^ (Re = Eu and Yb) and SrLa_2_Sc_2_O_7_:0.06Bi^3+^, Mn^4+^ was synthesized by the high-temperature solid-state method. High purity SrCO_3_ (99.99%), La_2_O_3_ (99.99%), Sc_2_O_3_ (99.99%), Bi_2_O_3_ (99.99%), Eu_2_O_3_ (99.99%), MnO_2_ (99.99%), and Yb_2_O_3_ (99.99%) were used as raw materials. The ingredients are mixed together thoroughly in an agate mortar for 10 min according to the stoichiometric proportions. The raw materials were then placed in a crucible and heated at 900 °C for 6 h, and then at 1500 °C for 6 h. Finally, by cooling the synthetic samples to room temperature, it was then ground into a powder for subsequent measurement.

Samples were analyzed by a Bruker D8 X-ray diffractometer (XRD) under Cu Kα radiation at 40 kV and 40 mA, with the radiation source parameters set to *λ* = 1.5406 Å, 2*θ* = 10–80°, and step sizes = 0.05 s/step. Rietveld structure were carried out using the General Structural Analysis System (GSAS) software (version 1251). The excitation and emission spectra were measured by a Japan Hitachi F-7000 fluorescence spectrometer; its resolution can reach 1 nm. The decay curves were recorded on a HORIBA FLuorolog-3 fluorescence spectrometer; the instrument can measure lifetime values from 10 ps to 10 s.

## 3. Results and Discussion

### 3.1. Phase Information

**Figure 1:**
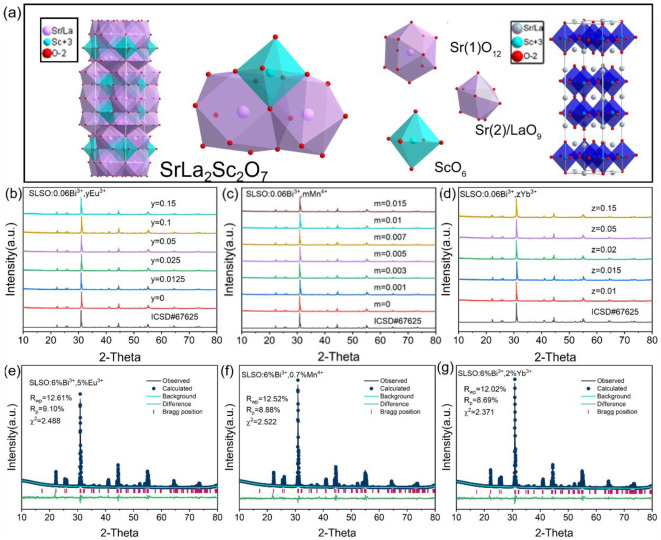
(**a**) Crystal structure of SrLa_2_Sc_2_O_7_; (**b**–**d**) XRD patterns of SLSO:0.06Bi^3+^, *y*Eu^3+^, SLSO:0.06Bi^3+^, *m*Mn^4+^, and SLSO:0.06Bi^3+^, *z*Yb^3+^; (**e**–**g**) Refined results of SLSO:0.06Bi^3+^, 0.05Eu^3+^, SLSO:0.06Bi^3+^, 0.007Mn^4+^, and SLSO:0.06Bi^3+^, 0.02Yb^3+^.

Generally, the performance of phosphor is affected by the crystal structure [23]. Figure 1a depicts the crystal structure of the SrLa_2_Sc_2_O_7_ unit cell and the crystal structure of the SrLa_2_Sc_2_O_7_ orthorhombic system and the *Pmmm* space group. It is revealed that the Sr1, Sr2/La, and Sc sites are surrounded by oxygen atoms with the coordination numbers of 12, 9, and 6, respectively. It is worth noting that the [Sr1O_12_], [Sr2O_9_] polyhedral and the [ScO_6_] octahedra are joined together by shared edges to form the basic unit. Figure 1b–d present the standard XRD pattern of SrLa_2_Sc_2_O_7_ (SLSO) (ICSD#67625), the XRD patterns of SLSO:0.06Bi^3+^, *y*Eu^3+^ (*y* = 0, 0.0125, 0.025, 0.05, 0.1, and 0.15), SLSO:0.06Bi^3+^, *m*Mn^4+^ (*m* = 0, 0.001, 0.003, 0.005, 0.007, 0.01, and 0.015), and SLSO:006Bi^3+^, *z*Yb^3+^ (*z* = 0. 01, 0.015, 0.02, 0.05, and 0.15). The Rietveld refinement of the XRD patterns obtained by slow sweeping using the GSAS program is required. Here, a series of SLSO: Bi^3+^, Eu^3+^, SLSO: Bi^3+^, Mn^4+^, and SLSO: Bi^3+^, Yb^3+^ was refined separately, and the diffraction peaks of all samples were basically consistent with the standard card (ICSD#67625). Figure 1e–g show the Rietveld refinement results for the representative samples of the Eu^3+^/Mn^4+^/Yb^3+^-doped SLSO: Bi^3+^. The black “-” indicates the intensity obtained from experimental tests, the blue dot represents the calculated intensity, the green line indicates the background, the green horizontal line indicates the error between the intensity obtained from experimental tests and the calculated intensity, and the plum-red “|” indicates the Bragg reflection position of the calculated pattern. The refinement parameters *R*_wp_ < 15%, *R*_p_ < 10%, and χ^2^ < 5 are within a reasonable range. These indicate that the Eu^3+^/Mn^4+^/Yb^3+^ and Bi^3+^ incorporation into the SLSO could maintain the phase purity and no impurity phase was generated at the current doping level.

### 3.2. Luminescence Properties of SLSO:0.06Bi^3+^, yEu^3+^

**Figure 2:**
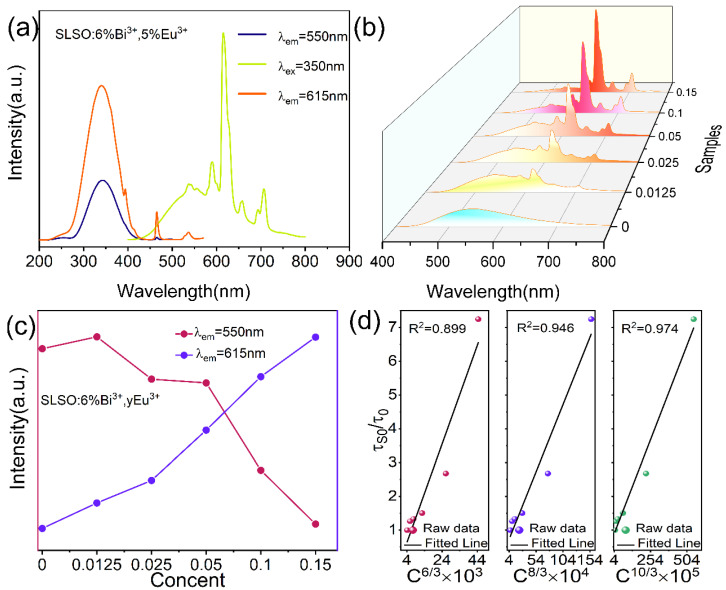
(**a**) Excitation and emission spectra of SLSO:0.06Bi^3+^, 0.05Eu^3+^; (**b**) emission spectra of SLSO:0.06Bi^3+^, *y*Eu^3+^ (*λ*_ex_ = 350 nm); (**c**) Emission intensities of SLSO:0.06Bi^3+^, *y*Eu^3+^ (*λ*_em_ = 550 and 615 nm); (**d**) relationship between τ_S0_/τ_0_ with C^6/3^, C^8/3^, and C^10/3^.

Figure 2a shows the emission and excitation spectra of SLSO:0.06Bi^3+^, 0.05Eu^3+^. For the 615 nm emission peak, the band centered at 350 nm, along with the characteristic absorption bands of Eu^3+^ ions at 394, 465, and 537 nm, which can be attributed to the ^7^F_0_-^5^L_6_, ^7^F_0_-^5^D_2_, and ^7^F_0_-^5^D_1_ of Eu^3+^ [24]. Moreover, the wide band is comprised of a ^1^S_0_→^3^P_1_ transition of Bi^3+^ ions and a charge transfer band (CTB) transition (Eu^3+^-O^2-^), while these sharp peaks are associated with the 4f-4f characteristic transitions of Eu^3+^ ions. Under the 350 nm excitation, as shown in Figure 2b, not only a broad emission band centered at 550 nm but also several sharp peaks at 581, 590, 598, 615, 658, and 707 nm were observed from the emission spectra of SLSO:0.06Bi^3+^, *y*Eu^3+^, which correspond to the ^5^D_0_→^7^F_J_ (J = 0, 1, 2, 3, 4) characteristic transitions of Eu^3+^ ions. Among them, the 590 and 598 nm emission peaks, corresponding to the ^5^D_0_→^7^F_1_ transition, are magnetic dipole transitions and are insensitive to positional symmetry in the crystal structure, while the red emission at 615 nm is attributed to the ^5^D_0_→^7^F_2_ electric dipole transition [25,26,27], which arises from the lack of inversion symmetry at the Eu^3+^ site; its emission intensity is much stronger than that of the transition ^7^F_1_ level. When Eu^3+^ ions are in a low-local symmetric environment, the ^5^D_0_-^7^F_2_ is dominant [28]. Considering the similar ionic radius and the same charge, Eu^3+^ ions tend to occupy the position of La^3+^ in SLSO. As can be observed in the spectrum, the ^5^D_0_-^7^F_2_ emission of Eu^3+^ at 615 nm is the strongest emission peak. In Figure 2c, with the increase in Eu^3+^ concentration from *y* = 0 to *y* = 0.15, the red emission intensity at 615 nm appears to increase significantly, while the yellow emission intensity at 550 nm is constantly decreased, which shows the energy transfer from Bi^3+^ to Eu^3+^. The critical distance (*R*_c_) is of great significance for evaluating the energy transfer mechanism from Bi^3+^ to Eu^3+^ in SLSO: Bi^3+^, Eu^3+^. The following equation can be used (see [29,30]).

(1) $$Rc\;\approx \;2(\frac{3V}{{4\pi x}_{c}N}{)}^{1/3}$$

where the *x*_c_ value is the critical concentration of dopant ions (total concentration of Bi^3+^ and Eu^3+^), referring to the luminescence intensity of Bi^3+^ in SLSO: Bi^3+^, Eu^3+^, which is half of the initial value luminescence intensity; *V* is the cell volume size of 682.987 Å^3^; *N* = 4. Therefore, the calculated *R*_c_ is 12.68 Å, excluding the exchange interaction mechanism (*R*_c_ ≈ 5Å). Thus, for SLSO: Bi^3+^, Eu^3+^, the electric multipole interaction is used for the energy transfer of Bi^3+^-Eu^3+^. The energy transfer equation for multipolar interactions was derived according to the theory of Dexter and Reisfeld [31].

(2) $$\frac{\tau_{S0}}{\tau_{S}}\propto C^{\alpha /3}$$

where C is the total concentration of Bi^3+^ and Eu^3+^ ions. *τ*_S_ and *τ*_S0_ represent the lifetime values of Eu^3+^-doped and non-Eu^3+^-doped, respectively. The values α = 6, 8, and 10 indicate different types of dipole–dipole, dipole–quadrupole, and quadrupole–quadrupole interactions, respectively. As visualized in Figure 2d, the fitting factors of SLSO:0.06Bi^3+^, *y*Eu^3+^ samples are equal to 0.899, 0.946, and 0.974 for α = 6, 8, and 10, respectively, where *R*^2^ obtains the maximum at α = 10; hence, the energy transfer mechanism of Bi^3+^-Eu^3+^ is quadrupole–quadrupole interaction.

**Figure 3:**
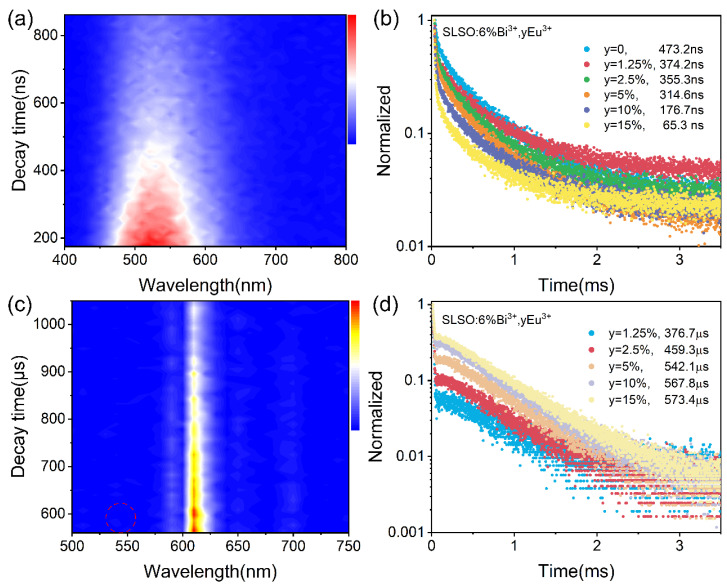
(**a**,**b**) Time-resolved spectrum and fluorescence decay curve of Bi^3+^; (**c**,**d**) time-resolved spectrum and fluorescence decay curve of Eu^3+^ in SLSO: Bi^3+^, *y*Eu^3+^.

Time-resolved photoluminescence (TRPL) spectroscopy reveals the dynamic behavior of doped ions with time in the emission spectrum, providing the ability to discriminate luminescent ions. Under the 350 nm Nano-LED lamp excitation, the TRPL spectra and fluorescence decay curves of SLSO:0.06Bi^3+^, 0.05Eu^3+^ are shown in Figure 3. Since the lifetimes of Bi^3+^ and Eu^3+^ are not on the corresponding level of magnitude, it can be noticed that the TRPL spectra of Bi^3+^ and Eu^3+^ do not appear at the same time in the coordinating system, as is visualized in Figure 3a,c. Figure 3b,d show the decay curves of Bi^3+^ and Eu^3+^ in SLSO:0.06Bi^3+^, *y*Eu^3+^, respectively, and the lifetimes are calculated as follows [32,33,34,35]:

(3) $${Ι(t)=A}_{1}{\exp(-t/\tau }_{1}{)+A}_{2}{\exp(-t/\tau }_{2})$$

where *t* represents the decay time, *τ*_1_ represents the fast decay lifetime, and *τ*_2_ represents the slow decay lifetime. The average lifetime of the activated ion (*τ*_av_) can be expressed by the following equation:

(4) $$\tau_{\mathrm{av}}{=(A}_{1}\tau_{1}{}^{2}{+A}_{2}\tau_{2}{}^{2}{)/(A}_{1}\tau_{1}{+A}_{2}\tau_{2})$$

The lifetimes of Bi^3+^ decrease from 473.2 to 65.3 ns with increasing Eu^3+^ concentration, while the lifetime of Eu^3+^ increases sustainably from 376.7 to 573.4 μs. This means there is an energy transfer from Bi^3+^ to Eu^3+^.

**Figure 4:**
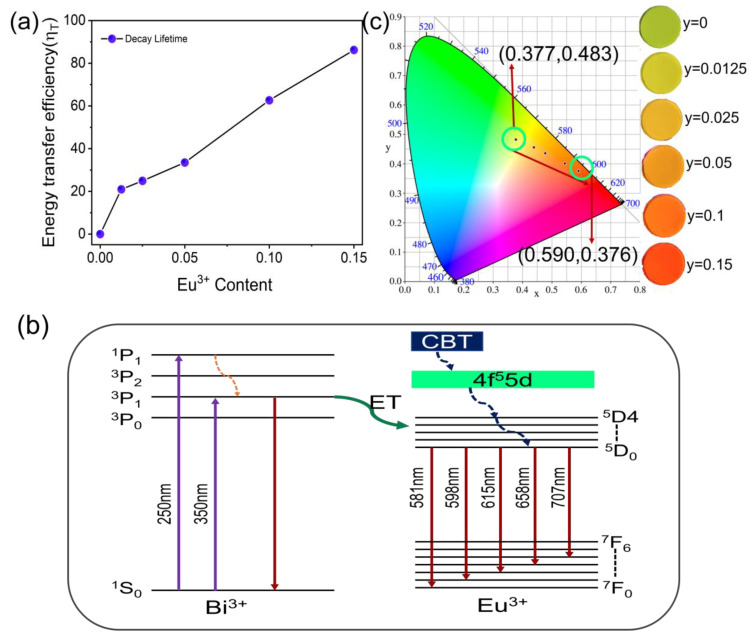
(**a**) Energy transfer efficiency of SLSO:0.06Bi^3+^, *y*Eu^3+^; (**b**) schematic energy level diagram of Bi^3+^ and Eu^3+^ in SLSO; (**c**) color coordinates and luminescence photos of SLSO: Bi^3+^, *y*Eu^3+^.

As shown in Figure 4a, when *y* is at 0.15Eu^3+^, energy transfer efficiency can reach 86.2%, and its value increases continuously with increasing Bi^3+^ concentration. This is because the emission peak of Bi^3+^ has an overlapping region with the excitation peak of Eu^3+^ to promote radiation reabsorption progress and, hence, energy transfer efficiency. Figure 4b illustrates the energy level diagram of Bi^3+^ and Eu^3+^ in SLSO. Figure 4c depicts the Commission International de L’ Eclairage (CIE) chromaticity coordinates of SLSO:0.06Bi^3+^, *y*Eu^3+^. Obviously, SLSO: 0.06Bi^3+^, *y*Eu^3+^ can emit yellow and red light, and the CIE values are from (0.377, 0.483) to (0.590, 0.376) with increasing concentrations of Eu^3+^.

### 3.3. Luminescence Properties of SLSO:0.06Bi^3+^, mMn^4+^

**Figure 5:**
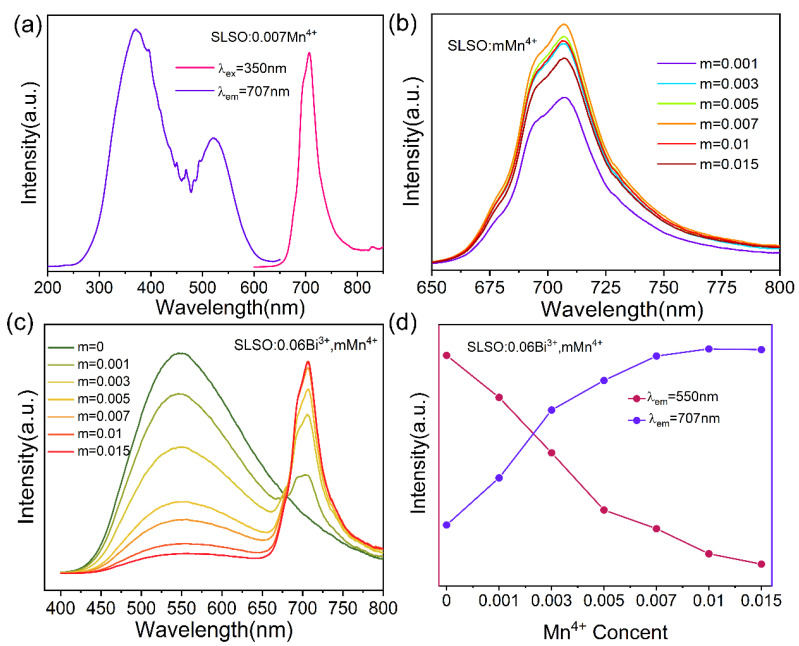
(**a**) Excitation and emission spectra of SLSO:0.007Mn^4+^; (**b**) emission spectra of SLSO: *m*Mn^4+^; (**c**) emission spectra of SLSO:0.06Bi^3+^, *m*Mn^4+^ (*λ*_ex_ = 350 nm); (**d**) the emission intensities of SLSO:0.06Bi^3+^, *m*Mn^4+^ (*λ*_em_ = 550 and 707 nm).

To further increase the luminescence intensity in the red region, we chose MnO_2_ as the raw material to provide Mn^4+^ ion co-doped with Bi^3+^ ion for luminescence, which provides the basis for the preparation of white LEDs with a high color rendering index and a low correlated color temperature. Figure 5a,b show the emission and excitation spectra of SLSO: *m*Mn^4+^ (*m* = 0.001, 0.003, 0.005, 0.007, 0.01, and 0.015). As the samples were prepared in air, it was speculated that the Mn ions were not reductive and we did not consider the red luminescence to be Mn^2+^. Secondly, most of the luminescence of Mn^2+^ is a green or red broadband spectrum, whereas the Mn luminescence is a narrow band from 650 to 800 nm in Figure 5a. Afterwards, to further figure out the Mn^4+^ luminescence center, we surveyed some published literature and found that the luminescence around 707 nm, with narrowband emissions, is that of Mn^4+^ ions. It can be seen that the excitation spectrum contains the 200–450 nm broadband, which is attributed to the charge migration band of Mn^4+^-O^2-^ and the ^4^A_2g_→^4^T_1g_ energy level transition of Mn^4+^; the weaker excitation peak at 395 nm is ascribed to the ^4^A_2g_→^2^T_2g_ transition of Mn^4+^; the other excitation peaks at 450–600 nm are considered to be the ^4^A_2g_→^4^T_2g_ energy level transition. SLSO: *m*Mn^4+^ presents the same spectral shape, and the emission intensity can reach a maximum at 0.007 Mn^4+^; there is a concentration quenching effect. Figure 5c shows that the emission spectra of SLSO: Bi^3+^, Mn^4+^ contain the emission peaks of Bi^3+^ and Mn^4+^, as shown in Figure 5d; with increasing Mn^4+^ concentrations, the emission intensities of Bi^3+^ decrease and the emission intensities of Mn^4+^ increase. This indicates that there may be an energy transfer from Bi^3+^ to Mn^4+^ in SLSO:0.06Bi^3+^, *m*Mn^4+^. In our previous research, it was found that Bi^3+^ ions enter the lattice sites Sr1 and Sr2(La) in SLSO; however, in this paper, the radius of Mn^4+^ ions r = 0.67Å (CN = 6) is similar to Sc^3+^ ion radius r = 0.745Å (CN = 6), which is suitable for Mn^4+^ ions to dope into. Therefore, it is believed that Mn^4+^ enters the [ScO_6_] octahedron.

**Figure 6:**
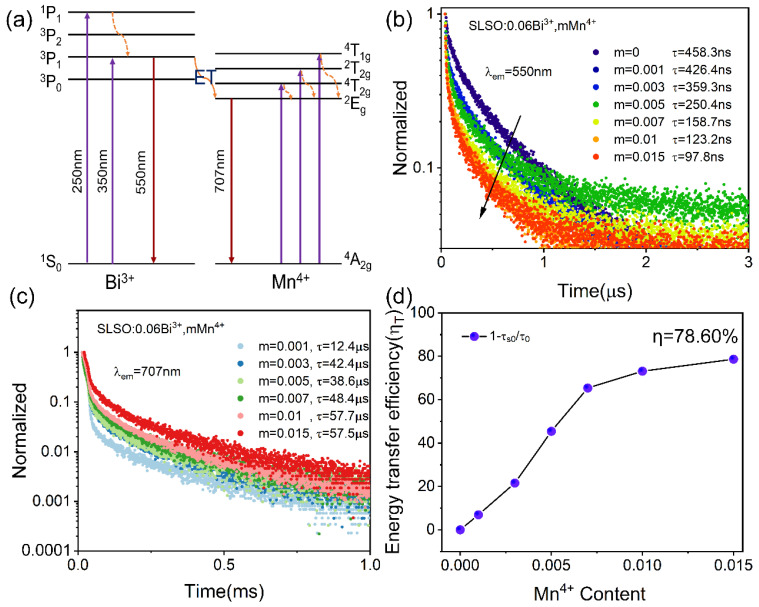
(**a**) Schematic energy level diagram of Bi^3+^/Mn^4+^ in SLSO; (**b**) the decay curves of Bi^3+^; (**c**) the decay curves of Mn^4+^; (**d**) energy transfer efficiencies of SLSO:0.06Bi^3+^, *m*Mn^4+^.

Figure 6a depicts the energy transfer process of Bi^3+^ and Mn^4+^, where the electrons were pumped to the excited states ^1^P_1_ and ^3^P_1_ from the ground state ^1^S_0_ under 250 and 350 nm excitation, according to the 6S^2^ outer-electron [36]. Meanwhile, the electrons on the ground state ^4^A_2g_ of Mn^4+^ were excited by the UV light source transition to the excited states ^4^T_1g_, ^2^T_2g_, and ^4^T_2g_ [37]. Then, the electrons relaxed to the lowest excited states ^3^P_1_ and ^2^E_g_ and released photons to emit 550 and 707 nm light through the radiation process back to the ground state. The variation in the fluorescence lifetime of the sensitizer ions doped in the matrix is direct evidence that energy transfer occurs. Figure 6b presents the decay curves of SLSO: Bi^3+^, *m*Mn^4+^. All decay curves can be well fitted to a double exponential function, and the lifetime values of Bi^3+^ were 458.3, 426.4, 359.3, 250.4, 158.7, 123.2, and 97.8 ns. Moreover, the decay curves of Mn^4+^ in SLSO:0.06Bi^3+^, *m*Mn^4+^ were also measured, and the lifetime values of Mn^4+^ increased from 12.4 to 57.5 μs, as displayed in Figure 6c. Moreover, the energy transfer efficiency can reach 78.60%, as shown in Figure 6d. In this process, although some energy is lost because of the non-radiative transition, it is clear that Bi^3+^-Mn^4+^ can still exhibit an efficient energy transfer, which is mainly attributed to the fact that Bi^3+^ and Mn^4+^ ions have not only the same excitation band but also the same radiative reabsorption process because the emission spectrum of Bi^3+^ and the excitation spectrum of Mn^4+^ have a large overlap region.

**Figure 7:**
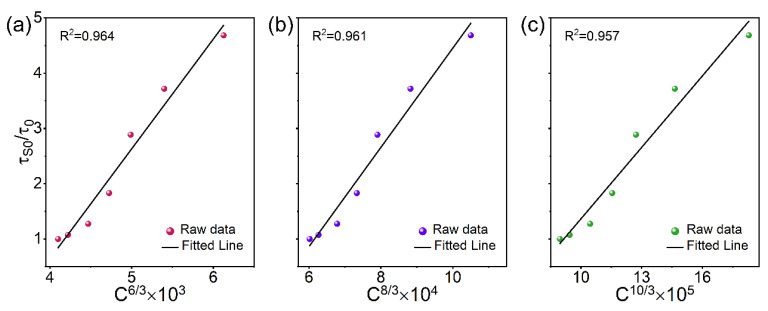
Relationship between τ_S0_/τ_0_ and (**a**) C^6/3^, (**b**) C^8/3^, and (**c**) C^10/3^.

The energy transfer process may be caused by the exchange interaction or the multipolar interaction; the former should be less than 5 Å, according to the critical distance (*R*_c_). The *R*_c_ value between Bi^3+^ and Mn^4+^ can be evaluated using Blasse’s theory [38], and the calculated *R*_c_ is 17.31 Å. It is proposed that the energy transfer mechanism may be the electron multipole–multipole interaction. In addition, the Dexter theory and the Reisfeld approximation can be utilized to analyze the multipole interaction type of the energy transfer mechanism. The values α = 6, 8, and 10 correspond to dipole–dipole, dipole–quadrupole, and quadrupole–quadrupole interactions, respectively. The plot of τ_S0_ /τ_0_ and C^α/3^ was fitted linearly, and the results are shown in Figure 7. By comparing the coefficient of determination (*R*^2^ value), the best fit was obtained for α = 6, and the outcome clearly illustrates that the energy transfer from Bi^3+^ to Mn^4+^ in SLSO is primarily defined by electric dipole–dipole interaction. The Mn^4+^ ion can emit strong deep red light, which can be excited in near ultraviolet for the lighting equipment of plant growth.

### 3.4. Luminescence Properties of SLSO:0.06Bi^3+^, zYb^3+^

**Figure 8:**
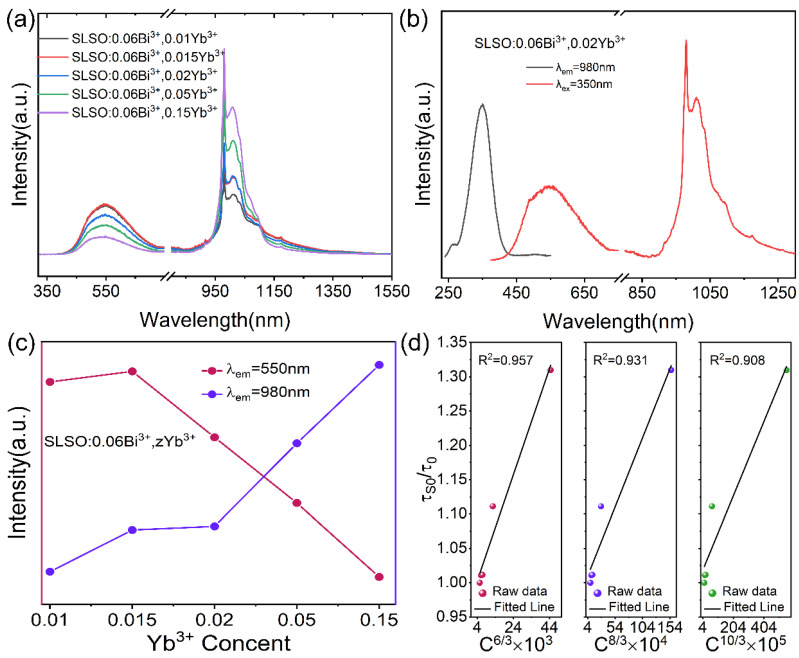
(**a**) Emission spectra of SLSO: Bi^3+^, *z*Yb^3+^; (**b**) excitation and emission spectra of SLSO: Bi^3+^, 0.02Yb^3+^; (**c**) emission intensities of SLSO:0.06Bi^3+^, *z*Yb^3+^ (*λ*_em_ = 550 and 980 nm); (**d**) relationship between τ_S0_ /τ_0_ and C^6/3^, C^8/3^, and C^10/3^ in SLSO:0.06Bi^3+^, *z*Yb^3+^.

In order to explore the possibility of near-infrared luminescence in SLSO, we co-doped Yb^3+^ with Bi^3+^. Figure 8a shows the emission spectra of SLSO:0.06Bi^3+^, *z*Yb^3+^ (*z* = 0.01, 0.015, 0.02, 0.05, and 0.15), which can range from 400 to 1350 nm under the 350 nm excitation. Figure 8b depicts the excitation spectrum and the emission spectrum of SLSO:0.06Bi^3+^, 0.02Yb^3+^, and the 980 nm emission peak reaches the high excited state ^2^F_5/2_ to ground state ^2^F_7/2_ of the Yb^3+^ ions [39]; the 550 nm emission peak is due to the excited state ^3^P_1_ to ground state ^1^S_0_ of the Bi^3+^ ions. As shown in Figure 8c, obviously, with an increase in Yb^3+^ concentrations, the emission intensities of 980 nm increase and those of 550 nm decrease; it means there may be an energy transfer from Bi^3+^ to Yb^3+^. According to the critical distance (*R*_c_) [40], for SLSO: Bi^3+^, Yb^3+^, *N* = 4, *V* = 683.702 Å^3^, and *x*_c_ = 0.11, the critical distance is *R*_c_ = 14.37 Å. The plot of τ_S0_ /τ_0_ and C^α/3^ is fitted linearly in Figure 8d, where α = 6, 8, and 10 corresponds to dipole–dipole (d–d), dipole–quadrupole (d–q), and quadrupole–quadrupole (q–q) interactions, respectively. The fitting factors of *R*^2^ are 0.957, 0.931, and 0.908, respectively, and the best fit was obtained for α = 6, showing that Bi^3+^ and Yb^3+^ belong to the d–d interaction.

**Figure 9:**
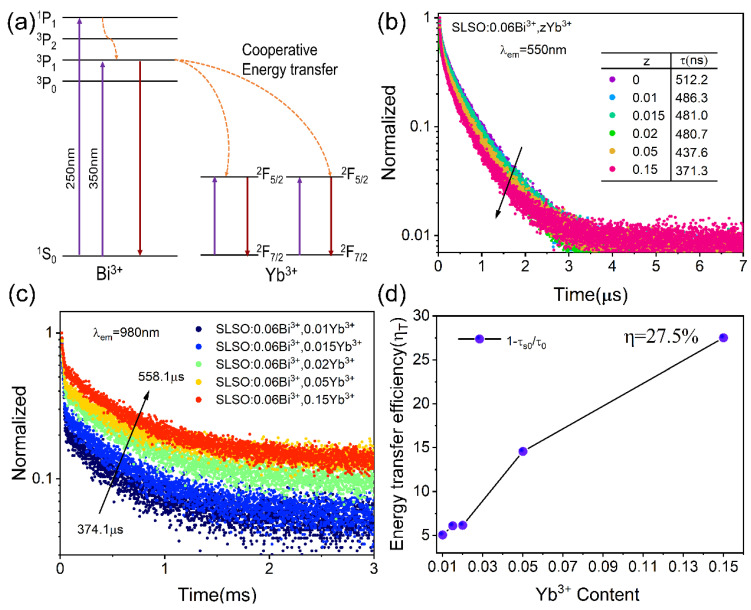
(**a**) Schematic energy level diagram of Bi^3+^ and Yb^3+^ ions in SLSO; (**b**) decay curves of Bi^3+^; (**c**) decay curves of Yb^3+^; (**d**) energy transfer efficiencies of SLSO:0.06Bi^3+^, *z*Yb^3+^.

Figure 9a depicts the energy transfer process of Bi^3+^-Yb^3+^ [41]. Figure 9b shows the decay curves of Bi^3+^ in SLSO: Bi^3+^, *z*Yb^3+^, and the lifetime values were 512.2, 486.3, 481.0, 480.7, 437.6, and 371.3 ns. Obviously, the lifetime of Bi^3+^ ions gradually decayed, and Figure 9c presents the lifetime values of a Yb^3+^ increase from 374.1 to 558.1 μs, which means the energy transfer from Bi^3+^ ions to Yb^3+^ ions. The energy transfer efficiency was calculated as follows: η = 1-τ_S0_ /τ_0_ [42], and Figure 9d shows that the η value can reach a maximum of η = 27.5% at *z* = 0.15. Since the excitation spectrum of Yb^3+^ ion is concentrated in the near-UV region and the emission spectrum of Bi^3+^ is in the visible region, they have fewer overlapping areas, so the energy transfer efficiency between them is low. However, it is found that the Bi^3+^-Yb^3+^ co-doping greatly broadens the emission range; this means that SLSO: Bi^3+^, *z*Yb^3+^ has a promising application in the visible illumination region as well as the near-infrared detection region.

## 4. Conclusions

In summary, a series of SLSO:0.06Bi^3+^, *y*Eu^3+^, SLSO:0.06Bi^3+^, *m*Mn^4+^, and SLSO:0.06Bi^3+^, *z*Yb^3+^ was synthesized by the high-temperature solid-state method and could create tunable color emissions by the energy transfer from Bi^3+^ to Eu^3+^/Mn^4+^/Yb^3+^, which was proven by the decay curves, time-resolved spectra, and spectral properties. The experiments have not only discussed the process of energy transfer from Bi^3+^ ions to other luminescent centers but have also investigated the interactions between the sensitized and activated ions. Based on the above studies, we believe that a large overlapping area between the emission spectrum of the sensitizer and the excitation spectrum of the activator will result in high energy transfer efficiency. Therefore, in order to prepare multifunctional luminescent materials with both visible and near-infrared emissions, the intense luminescence of the Yb^3+^ ion needs to be improved so that the emission spectrum of Bi^3+^ can be modulated to blue-shift to get more overlapping regions in the future in order to obtain efficient energy transfer progress between Bi^3+^ and Yb^3+^, which can provide a novel tunable wavelength luminescent material for high-quality lighting and detection applications.

## Data Availability

Not applicable.
